# Exploring the Molecular Space of Bitter Peptides via Sensory, Receptor, and Sequence Data

**DOI:** 10.1021/acs.jafc.5c01195

**Published:** 2025-07-23

**Authors:** Alexandra Steuer, Laura Sophie Eckrich, Silvia Schaefer, Verena Karolin Mittermeier-Kleßinger, Alexander Otterbach, Maik Behrens, Corinna Dawid, Antonella Di Pizio

**Affiliations:** a TUM Graduate School, TUM School of Life Sciences, 9184 Technical University of Munich, Alte Akademie 8, Freising 85354, Germany; b Leibniz Institute for Food Systems Biology at the Technical University of Munich, Freising 85354, Germany; c Professorship for Functional Phytometabolomics, TUM School of Life Sciences, 9184 Technical University of Munich, Freising 85354, Germany; d Chair of Food Chemistry and Molecular Sensory Science, TUM School of Life Sciences, 9184 Technical University of Munich, Freising 85354, Germany; e Professorship for Chemoinformatics and Protein Modelling, TUM School of Life Sciences, 9184 Technical University of Munich, Freising 85354, Germany

**Keywords:** bitter peptide, taste threshold, peptide sequence, bitter taste receptors, food

## Abstract

This study explores the chemical space of bitter peptides through a curated data set, named Bitter Peptide Space (BPS)-1000, which includes experimentally validated bitter and nonbitter peptides. The data set integrates sensory data, bitter taste thresholds (BTTs), and bitter taste receptor (TAS2R) activity when available. The inclusion of modified peptides further expands the data set’s diversity. The HELM (Hierarchical Editing Language for Macromolecules) and BILN (Boehringer Ingelheim Line Notation) notations have been generated to provide a unique representation for both canonical and modified peptides. Through sequence-based and structure-based analyses, the study highlights the role of hydrophobicity, molecular size, and specific amino acid composition in the bitter and nonbitter sets in canonical and modified peptides, suggesting differences that could contribute to bitterness and enhancing the understanding of bitter peptide characteristics.

## Graphical abstract

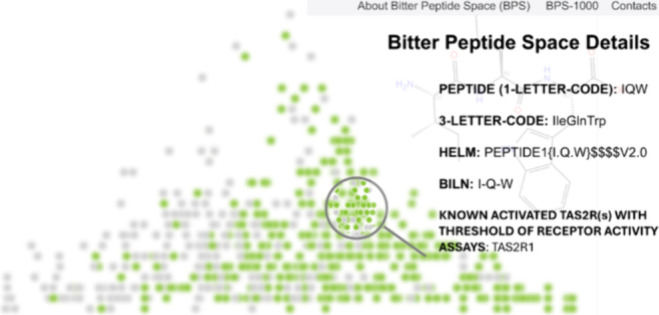

## Introduction

Although aided by visual inspection, 86% of consumers profess that flavor, as a food-quality attribute, is the most important criterion for their decisions when purchasing any kind of food or beverage.^1^ Flavor is defined as the interplay between aroma, taste and trigeminal-mediated chemosensations. Five basic taste qualities, for example, allow humans to evaluate the energy content available in the form of carbohydrates (sweet taste), proteins and amino acids (umami taste), potentially harmful food components (bitter and, to a lesser extent, sour tastes) and table salt, which affects our body’s electrolyte balance (salt taste).^2^

Due to the growing world population and individual consumer needs, the global demand for sustainable, nutritious, functional and at the same time tasty food proteins increased during the past decade.^3^ Proteins belong to the three macronutrients and hence, are an important source of energy.^4^ Although, besides animal-based proteins, plant-based protein concentrates, isolates, and hydrolysates exhibit promising nutritional and techno-functional properties, for several food applications their use is often hindered by sporadic off-flavor notes.^5^ Especially secondary metabolites, peptides and free amino acids, noncovalently binding to the proteins, exhibit a long-lasting bitter off-taste, which often leads to consumer complaints.^6^

A large variety of peptides, as well as essential branched amino acids, trigger aversive bitter taste perception.^7^ While in animal-based foodstuffs and beverages, such as cheese, bitter peptides mainly formed during processing and fermentation rarely exhibit off-notes, they play a major role in the unpleasant bitterness of plant-based protein (partial) hydrolysates and fermentates. For example, whey, pea, potato, canola and soy protein hydrolysates are well-known for the presence of bitter-tasting peptides.^8^

Bitterness appears to be an inherent consequence of proteolysis.^9,10^ Different proteolytic enzymes are implicated in bitterness development, including microbial activity by lactic acid bacteria, proteases, and peptidases.^9,11–13^ Consequently, enzymatic debittering strategies using exopeptidases, especially when used concurrently or sequentially with endopeptidases, have been proposed.^14–17^ Particularly, proteases that either reduce peptide size or selectively cleave at hydrophobic amino acids have proven particularly effective in debittering.^18–20^ However, these strategies do not universally succeed, as no single approach is effective across all substrates or processing conditions.^14,21^ Improving the bitter taste characteristics is particularly difficult because of the complexity of peptide sequences and the unresolved challenge of establishing a clear relationship between peptide sequence and taste function.

Hydrophobicity has been pointed out as a relevant molecular feature for determining bitterness since 1971, when Ney formulated the *Q*-value, based on free energy transfers (*Q* = ∑Δ*f*/*n*), as an estimation of peptide bitter taste.^22^ However, it is now clear that bitterness prediction is far more complex, requiring more sophisticated models and a deeper understanding of bitter peptides.^23–28^

To date, identified bitter peptides were collected and two data sets have been released. The BTP640 data set, with 320 bitter and 320 potential nonbitter peptides, was built by Charoenkwan et al. in 2020.^27^ The bitter data set was assembled with known canonical bitter peptides from the literature, while the nonbitter data set was assembled from the BIOPEP database of bioactive peptides with unknown bitter activity. Recently, the BTP640 was enlarged with additional bitter peptides to reach 360 bitter and 360 potential nonbitter peptides (i.e., BTP720).^24^

The collection of data on bitter peptides is challenged by the inherently complex nature of sensory data. In this work, we aimed to generate a manually curated database of bitter peptides that represents the current state of the art on bitter peptides. We have assembled the largest set of peptides for which we have experimental information on both bitter and nonbitter taste. This allowed us to provide a comprehensive investigation of the molecular chemical space of bitter peptides and navigate for sensory intensities (bitter taste thresholds, BTTs) and the knowledge of cognate receptors.

## Materials and Methods

### Preparation of Database

To create a valid prediction for bitter peptides, a data set containing 20 amino acids and 973 peptides was compiled based on published literature, patents and an in-house collection from the Chair of Food Chemistry and Molecular Sensory Science at the Technical University of Munich (TUM). Following the standard definition, when a sequence has a length <100 amino acids, we consider it a peptide. A prerequisite for including a peptide in the database was the availability of sensory data obtained with the synthesized or isolated peptides. Five peptides for which only functional data were available were also included. The amino acids of the peptides were present in the L configuration unless otherwise stated. The database consisted of 570 bitter peptides and 423 nonbitter peptides. A peptide was described by the intrinsic taste attributes bitter and nonbitter, where the attribute nonbitter can describe either taste active or not taste active peptides. If authors used taste qualities other than these five basic taste attributes, e.g., earthy or burning, they were not listed in the database. Parameters of interest were the following: peptide sequence, sequence length, taste quality, BTT, and activated receptor(s). If receptor assay data was available for some peptides but no sensory data, the “not tested” label was assigned to the sensory column.

### Peptide Representation

HELM (Hierarchical Editing Language for Macromolecules)^29^ and BILN (Boehringer Ingelheim Line Notation)^30^ notations were generated for all peptides. The HELM notation provides information on complex or simple polymers and monomers. Monomers are described as short unique identifiers, the atom/bond representation as building blocks, and both are stored in the HELMCoreLibrary and provided within the HELM monomer guidelines (https://pistoiaalliance.atlassian.net/wiki/spaces/HELM/pages/2534506549/HELM+Monomers). With the help of linear monomers, simple polymers are built. By combining additional information about hydrogen bonds, annotations, and simple polymers, the complex polymers can be generated. We used open-source tools available to enable the creation of HELM strings and a HELM toolkit for basic calculations (http://webeditor.openhelm.org/hwe/examples/App.htm;https://github.com/PistoiaHELM/HELMNotationToolkit). The BILN notation converts the atomic description into the line notation, by using a monomer library composed of a chemical structure like HELM and additionally, an identifier and attachment points that represent additional chemical entities “R”. By adding a unique identifier, BILN modifies the monomer information given in HELM notations and defines itself as a subset of the original definition of HELM. Two Python-based packages were used to work with the BILN notation: 1-one to convert HELM/BILN notations (https://github.com/rochoa85/BILN-converter), 2-one to create 2D/3D peptide structures molecules using the FASTA, HELM, or BILN notations (https://github.com/Boehringer-Ingelheim/pyPept). The generated dictionary is available at https://github.com/dipizio/BPS1000).

### Bitter Peptide Space Analysis

The analyses of the peptides were performed with KNIME (“Konstanz information miner”), an open-source platform for data processing and analysis, originally developed by the University of Konstanz (https://www.knime.com). KNIME extensions were integrated for individual and specific functions, e.g. to calculate molecular descriptors of chemical substances or their graphical representation (like with Vernalis or CDK) or the Python environment for visualization or customized evaluations. With the help of these tools, we established a self-constructed KNIME workflow that is uploaded and public available at our Github page (https://github.com/dipizio/BPS1000).

The first step of the analysis was the preparation of the BPS-1000 data set by integrating and reorganizing the relevant information into a standardized and usable format (Figure S1). This step was essential for extracting important information, such as the consistency of the taste annotations, and for transforming raw data, such as converting peptide sequences from three-letter to one-letter amino acid codes.

The KNIME workflows are publicly available as semiautomated tools to ensure reproducibility and transparency in our chemical analyses, even for users without a coding background. All parameters used are stored within the workflow and remain fully visible and accessible upon download.

The workflow is divided into two main parts. Part 1: “Analysis of Peptide Datasets” focuses on analyzing the bitter and nonbitter sets of collected peptides. First, the workflow splits peptides as canonical and noncanonical/modified. This is achieved using the *Row Splitter* node, where pattern matching is applied to the “canonical” column (with the condition set to “1” for canonical peptides). Then, the canonical peptides are analyzed based on sequence length. The *String Manipulation* node is employed for this task, utilizing KNIME’s built-in string length function to calculate peptide lengths. Following this, several *Data Preparation* nodes are used to format the table appropriately, enabling effective visualization. A bar chart is then generated, where the *x*-axis represents peptide lengths, and the bar colors distinguish between bitter (green) and nonbitter (blue) peptides, providing insight into sequence distribution and amino acid composition across the data set.

Part 2: “Analysis of chemical descriptors” focuses on the analysis of the physicochemical properties of peptides. Workflow 2 is designed to facilitate descriptor analysis through a two-branch pipeline. The first branch calculates *Q*-values based on the peptides’ one-letter code using the *Math Formula* node. The second branch computes molecular descriptors using the *RDKit Descriptor* node (RDKit: Open-source cheminformatics. https://www.rdkit.org). In this part of the workflow, SDF files containing the peptide structures are imported, and key descriptors—such as Exact Molecular Weight (ExactMW) and SlogP—are calculated and reported as numerical values. The partition coefficient logP is a widely accepted measure of hydrophobicity, defining the solubility ratio in two immiscible liquids like 1-octanol and water, resulting from experimentally or computationally determinations. While the experimental measurement requires higher expenses and duration, latterly developed in-silico approaches calculate the logP by adding the contribution of specific functional groups or atoms fragmented of a chemical molecule. The SlogP descriptor in RDKit is derived from the atom-type classification model developed by Wildman and Crippen, which estimates logP values based on additive contributions from predefined atomic properties.^31^

After completing both analytical workflows, we integrated the results and assigned color labels to each data point to aid in the visual interpretation of the scatterplots. Specifically, we used green to indicate bitter peptides and blue for nonbitter ones, applying the color manager for consistent labeling. Taste quality served as the categorical color dimension, while the *x*- and *y*-axes plotted hydrophobicity measures (logP or *Q* value) against molecular weight (ExactMW or peptide length). The statistical visualizations (Figures S2 and S3) were generated using the Python libraries Matplotlib and Seaborn, which provided high-quality, customizable plotting tools for our data set.^32,33^

## Results and Discussion

In this paper, we present a database of sensory-tested bitter and nonbitter peptides (Figure 1). Since it represents the chemical space covered by the collected bitter and nonbitter peptides (ca. 1000), we call it Bitter Peptide Space (BPS)-1000. Each entry of the database is associated with sensory and in vitro receptor activity data when available, with corresponding references, allowing users to trace back to the original sources. Importantly, many peptides yield inconsistent sensory results across different studies, and relying only on automated data collection could compromise the reliability of the information gathered in the data set. To address this issue, we have flagged entries with conflicting sensory data and cited the relevant literature sources reporting these discrepancies. This approach allows users to make informed decisions about using the data based on their specific application. For instance, computational researchers may choose to exclude inconsistent entries when training predictive models, while experimentalists may use these annotations to revisit and clarify discrepancies through further testing. Data is made available at https://bps1000.leibniz-lsb@tum.de to ensure easy data searchability.

**Figure 1:**
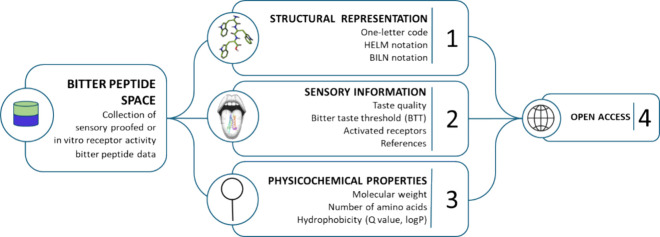
Schematic representation of the BPS-1000 data set and collected information.

### Composition of BPS-1000

The bitter and nonbitter peptides were selected from the literature with the precondition that their bitter taste was experimentally validated by human sensory or receptor studies. We gathered 993 sensory-tested peptides from about 162 peer-reviewed papers as well as 83 peptides from patents and in-house collection from the Chair of Food Chemistry and Molecular Sensory Science at TUM.

Collected bitter peptides (570) were found in about 255 food sources, which can be divided into four groups: animal-, plant-, yeast products and mixed products, i.e. these bitter peptides were found in plant products and animal products or in animal products and yeast products. Each group comprises 121, 102, 11, or 12 bitter peptides, respectively (Figure 2). The primary source of bitter peptides encloses animal product sources like cheese (e.g., Gouda, Cheddar, Cream cheese, Parmesan, etc.). Bitter-tasting plant-derived peptides are found in soya products or unprocessed or processed cocoa beans.

**Figure 2:**
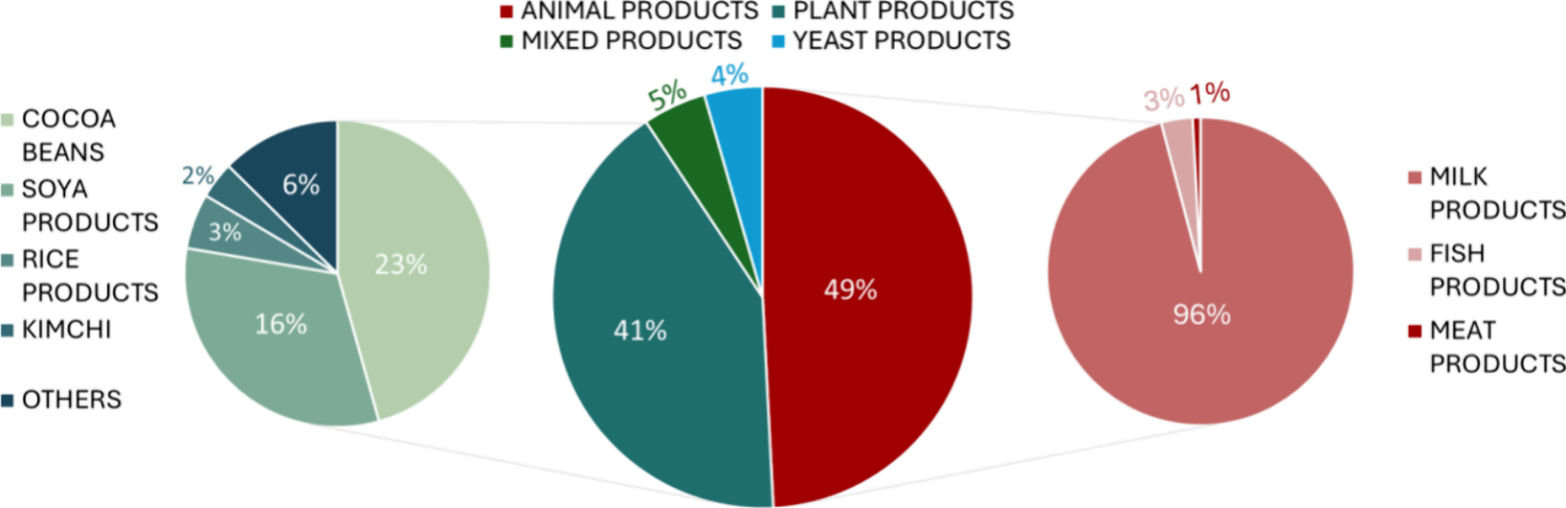
Food sources of bitter canonical peptides in BPS-1000. The central pie chart shows the principal food sources. The left pie chart divides the plant-derived food into specific components. The right pie chart illustrates up-to-date research on milk-derived peptides, including related milk proteins like α, β, and κ-casein and a small percentage of peptides derived from fish and meat products.

Most collected peptides were identified by activity-guided fractionation combined with analytical sensory tools like the taste dilution analysis,^34^ or, more recently, combining proteomics tools with sensory analytics.^35^ For 341 bitter peptides, we could also retrieve bitter taste thresholds (BTTs). The most bitter peptide is RRPPPFFF, with a BTT of 0.002 mmol/L, while the highest BTT values are 403 and 519 mmol/L for the dipeptides GE and GF. Reported BTTs for 86.1% of bitter peptides range from 0.002 mmol/L to 10 mmol/L.

Importantly, the sensory-validated set of nonbitter peptides (423) offers an opportunity to develop accurate models for bitter taste prediction. Accurate prediction of bitter peptides will be of value for tailoring food processing steps better in order to improve consumer acceptance, avoid food waste and to facilitate usage of side-streams.

### Standardized Representation for Both Canonical and Modified Peptides

Peptides in the BPS-1000 database are represented using peptide-specific notations, going beyond the commonly used one-letter code (FASTA format).

Each peptide is also provided with a FASTA notation. The standard FASTA format is limited to natural amino acids and does not accommodate modifications or cyclic peptides. To address this, we adopted common strategies for annotating nonstandard features, such as indicating d-form amino acids with a “D” (e.g., d-Leucine is noted as (D)-L). Modifications are included in brackets surrounding the amino acid. However, these adaptations do not provide a standardized representation that is unique for both canonical and modified peptides.

The correct conversion between biological and chemical representation, i.e. amino acid sequences vs chemical structure representations, is challenging. Recent efforts are being made to improve these representations. Zhang et al. developed the HELM (Hierarchical Editing Language for Macromolecules) notation to provide a transparent and traceable language for complex biomolecules like antisense oligonucleotides, peptides and proteins.^29^ HELM allows for the detailed representation of noncanonical amino acids, post-translational modifications, and chemical modifications that cannot be captured by the simple one-letter codes used in FASTA. HELM can also represent branched structures, cyclic peptides, and conjugates, making it more versatile for complex macromolecules. HELM is designed as a standardized notation that can be used across different software platforms and databases, ensuring that complex molecules are described consistently. This is especially useful for bioinformatics analyses where consistent, unambiguous representation is critical. Therefore, the HELM notation is now integrated into bioinformatics/chemoinformatics software (e.g., KNIME, https://www.knime.com/blog/accessing-the-helm-monomer-library-with-knime) and databases (e.g., ChEMBL, https://www.ebi.ac.uk/chembl/). The BILN (Boehringer Ingelheim Line Notation) notation was developed by Fox and colleagues (2022) to allow for an improved readable line format with respect to the HELM notation.^30^ As in HELM, the atomic description uses a monomer library defined by a chemical structure format (e.g., SMILES or an SDF MolBlock), but the rules for connections make the string readable and easy to generate.

Here, we provide HELM and BILN notations for all the BPS-1000 peptides. To generate the BILN notation of our data set, we have extended the monomer library initially developed from the Boehringer Ingelheim library with additional 36 monomers (the entire monomer library is available at https://github.com/dipizio/BPS1000).

### Bitter Peptides and Amino Acids Discovered by Functional Heterologous Receptor Assays

Humans perceive thousands of bitter compounds through ∼25 G protein-coupled receptors, the TAS2Rs (taste receptors type 2).^36–39^ In the oral cavity, the bitter taste receptors are expressed in bitter taste receptor cells.^40^ Though the expression of TAS2Rs is not confined to the oral cavity, it also occurs, e.g., in the intestinal and respiratory tracts as well as bladder epithelial cells. Hence, a wide variety of physiological functions, in addition to gustation, are assumed for bitter taste receptors.^41,42^

Functional heterologous expression of human TAS2Rs has contributed substantially to the discovery of TAS2R-activating bitter substances.^43^ However, so far, only a few studies included or focused on bitter peptides and amino acids as ligands for TAS2Rs. The very first report on the activation of human TAS2Rs by bitter peptides and amino acids was published by Maehashi and colleagues.^44^ Using fractionated trypsin-hydrolyzed casein as source for potential TAS2R stimulating agonists, it was reported that human TAS2R1, −4, −14, and −16 responded to the tested bitter fraction. To identify individual peptides that elicit responses, the dipeptides GL, GF, as well as the nonbitter GG were tested individually with cells expressing one of the four identified receptors. TAS2R1 exhibited strong responses to the two bitter peptides GL and GF but not to the nonbitter GG. TAS2R4, −14, and −16 showed signals below the chosen positive controls existing at this time. Therefore, the peptides could represent partial agonists for these targets, or, alternatively, since the positive control stimuli also showed superior potencies compared to the peptides, the limited potency may have prevented testing of higher doses and, hence, observation of higher signals as well. Of note, is the fact that in this study not all TAS2Rs were screened, but all 4 screened receptors were suggested to respond to GF, which may indicate that false-positive results cannot be fully excluded. In 2010, Upadhyaya and colleagues tested a number of di- and tripeptides against TAS2R1.^45^ The most potent TAS2R1 agonist found in their study was FFF, whereas the additional three employed dipeptides as well as the three additional tripeptides exerted medium potencies. Again, mock-control experiments to demonstrate the receptor-specificity of the activation were not shown. In a later study, F, P, R, FF, PR, and FFPR were screened as stimuli on TAS2R8 and TAS2R39 expressing cells.^46^ Significant responses were observed for FFPR with TAS2R8 and TAS2R39, whereas TAS2R39 also exhibited weaker responses also to PR. The report by Ueno and colleagues also reported the absence of FFPR responses in cells expressing the other TAS2Rs, which indicates the receptor-specificity of their observations.

In 2013, a very comprehensive study reported the screening of human TAS2Rs with all 20 biogenic L-amino acids and several peptides.^7^ In total five TAS2Rs exhibited responses, 3 of those, TAS2R1, −4, and −14 were reported by Maehashi and colleagues, whereas two additional receptors, TAS2R39 and −46 were found responsive. This study also included two complex peptides originally isolated from cheese,^47^ which were found to activate both TAS2R1 and TAS2R39. The tripeptide WWW was found as the most potent and universal agonist for the five identified TAS2Rs. Recently, an overlapping activation profile of WWW and bile acids at this subset of five TAS2Rs has been discovered, confirming the broad activity of the peptide WWW.^48^ The most limited activation spectra, responding only to WWW, were observed for TAS2R14 and TAS2R46. Interestingly, TAS2R4 and TAS2R39 also responded to d-Trp.

A subsequent report by Bassoli and colleagues found a somewhat larger array of TAS2Rs responding to W and F. Both stereoisomers of the two amino acids were used to screen the human TAS2Rs. Whereas the apparent stereoselectivity of TAS2R39 for d-Trp observed by Kohl et al. was not evident, the absence of selectivity of TAS2R4 for l-Trp and d-Trp was confirmed. For l-Trp additional weak responses were observed with TAS2R20 (former gene symbol TAS2R49) and TAS2R43. The screening with l-Phe resulted in the identification of four responsive TAS2Rs, TAS2R1, −4, −8, and −39. While all four receptors were activated by l-Phe, d-Phe elicited weak responses only in TAS2R1 and TAS2R39 expressing cells. As the absence of signals was not controlled with mock-transfected cells in this study, it cannot be excluded that some of the reported activities might have arisen independent from TAS2Rs.

Recently, Lang and colleagues investigated the TAS2R activating properties of cyclic peptides isolated from linseed oil, the so-called cyclolinopeptides (CL).^49^ The screening of 25 human TAS2Rs with five of the six classes of cyclolinopeptides revealed the activation of TAS2R14 and TAS2R43 expressing cells. Whereas only a single cyclolinopeptide of the CL6-class elicited responses in TAS2R43 expressing cells, TAS2R14 responded to CL1, CL2, CL3, CL4, and CL6 cyclolinopeptides. Interestingly, responses were only evident if at least one oxidized methionine residue was present in these peptides, which may indicate that neither the primary sequence nor the unmodified amino acids/peptides were detected. This suggests that these cyclic peptides may interact with the two TAS2Rs via a mechanism different from the previous amino acids/peptides.^49^

Activity data, summarized in Table 1 and Table S1, is available at https://bps1000.leibniz-lsb@tum.de.

**Table 1:** List of Amino Acids/Peptides Activating Human Bitter Taste Receptors^a^

TAS2R	amino acids, peptides	refs
TAS2R1	F, GF, GL, IF, LW, FI, FL, WW, GLL, IQW, LKP, FFF, WWW, YPFPGPIHNS, LVYPFPGPIHN	^7,44,45,48,50^
	(D)-F	
TAS2R39	F, W, IF, LW, PR, WW, WWW, FFPR, YPFPGPIHNS, LVYPFPGPIHN	^7,46,48,50^
	(D)-F, (D)-W	
TAS2R4	F, W, GF, GL, IF, LW, FW, WL, WF, WP, WW, LLL, WWW	^7,44,48,50^
	(D)-W	
TAS2R14	GF, WWW	^7,44,48,49^
	CL1, CL2, CL3, CL4, CL6	
TAS2R43	W	^49,50^
	CL6	
TAS2R8	FFPR	^46,50^
TAS2R16	GF	^44^
TAS2R20	F	^50^
TAS2R46	WWW	^7,48^

a CL, cyclolinopeptide. In Table S1, we report available EC_50_ values.

### Exploring the Bitter Peptide Space

To characterize the diversity of the peptides collected in BPS-1000, we present a comprehensive sequence-based analysis of these peptides. By using sequence data, we can explore the distribution of amino acids, peptide lengths, and physicochemical properties. Including nonbitter peptides in this collection is particularly important, as it allows us to explore how the bitter and nonbitter subsets differ.

Canonical peptides in BPS-1000 are composed of 20 different amino acids and have different lengths, with the longest peptides of 49 and 56 amino acids for the bitter and nonbitter sets, respectively (Figure 3A). Dipeptides are the most represented, with 107 bitter-tasting dipeptides and 71 nonbitter dipeptides. The distribution of amino acids is reported in Figure 3B. The amino acids F, G, I, L, P, R, V, W, and Y occur more in bitter peptides, whereas amino acids like A, D, E, M, S, and T are more frequent within nonbitter peptides. P is the most represented amino acid in the data set, it is found 241 times in nonbitter peptides and 569 times in bitter peptides.

**Figure 3:**
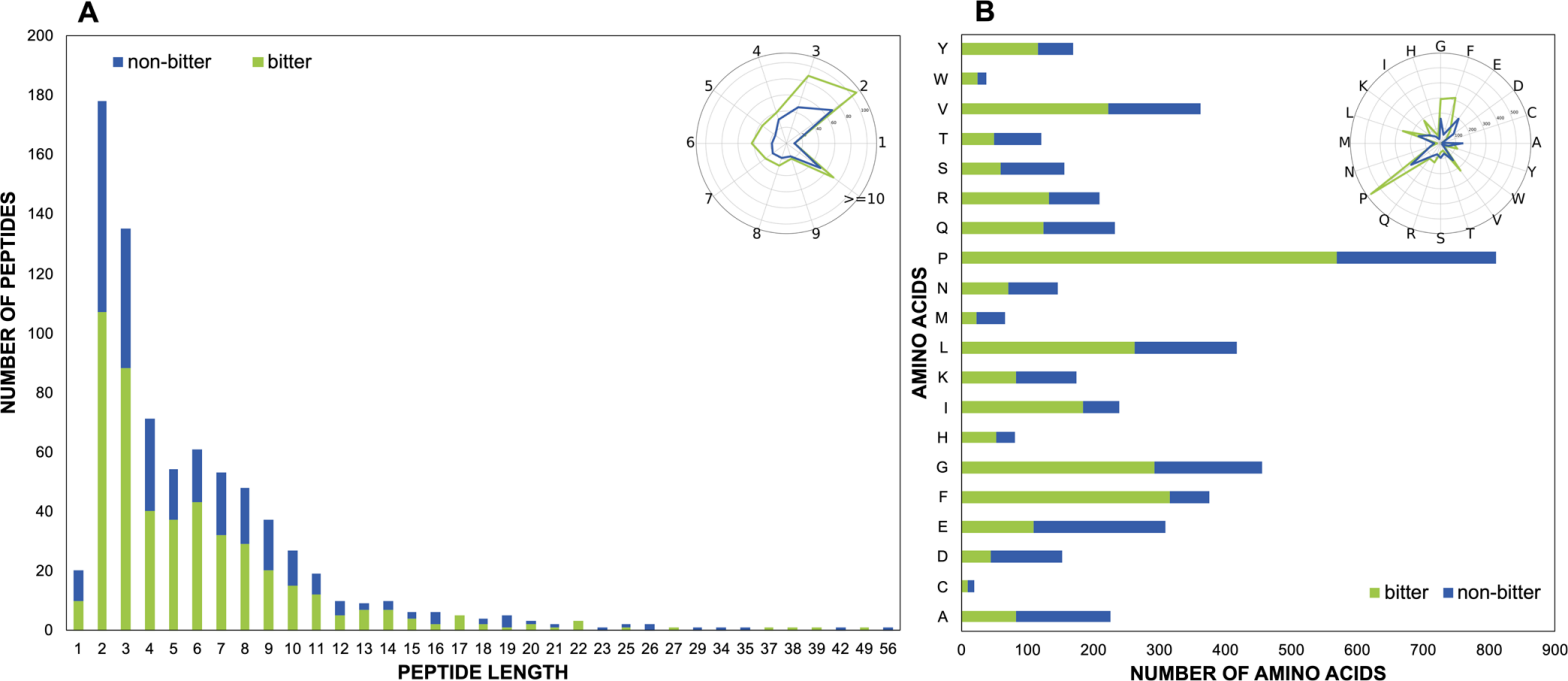
A) BPS-1000 canonical peptides by peptide length. Number of bitter (green bars) and nonbitter (blue bars) canonical peptides by length. B) Distribution of the individual amino acid composition in bitter and nonbitter canonical peptides within the BPS-1000.

To have a deeper view of the differences between the bitter and nonbitter sets of BPS-1000, we looked at their size and hydrophobicity. Previous structure–activity relationship studies have proved that steric or spatial structure features and hydrophobicity descriptors correlate with the bitter taste of peptides.^22,25^ We therefore used size and hydrophobicity to explore the peptide space of the BPS-1000 (Figure 4).

**Figure 4:**
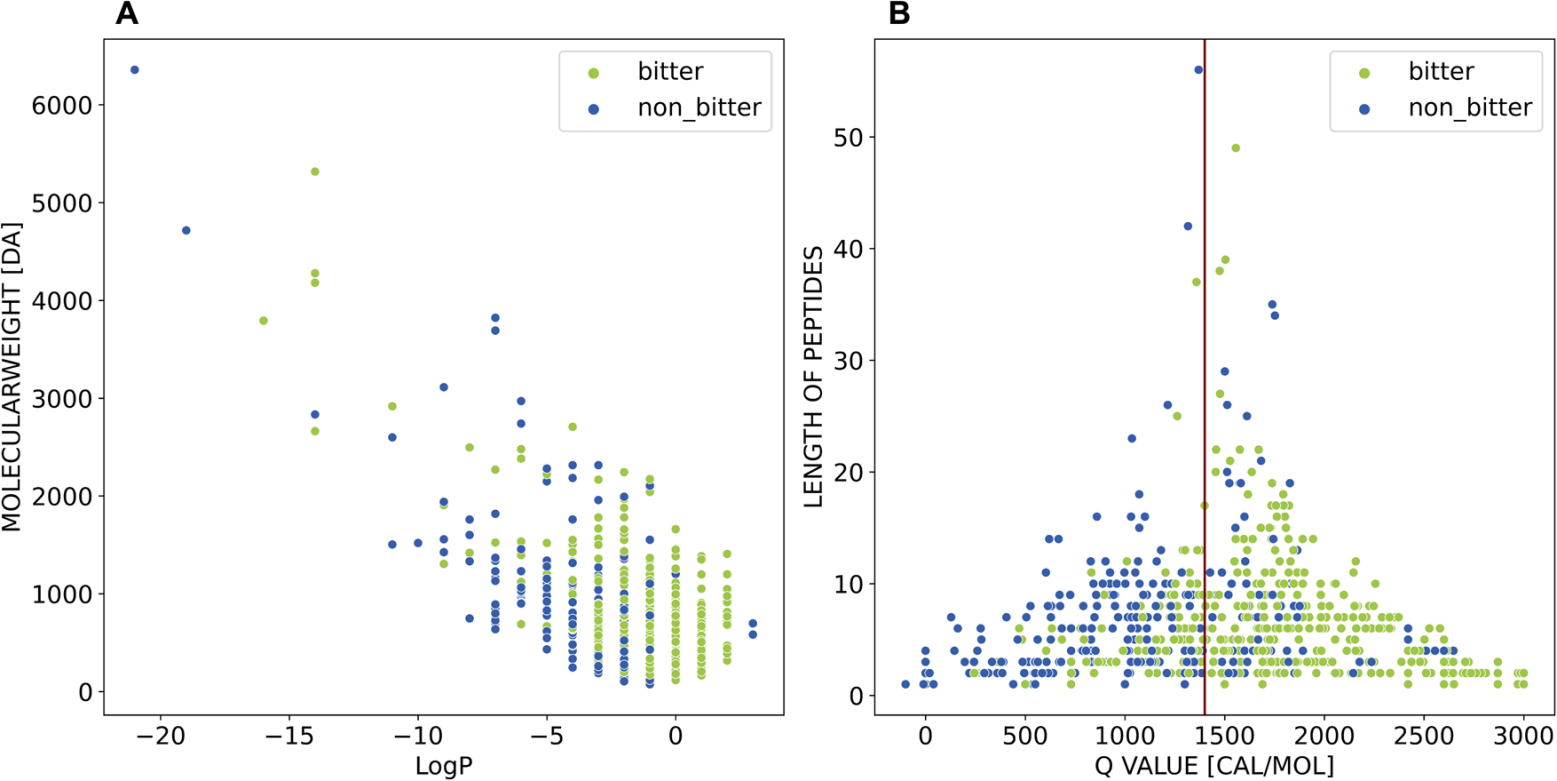
Peptide space investigation of the BPS-1000. A) Scatterplot of logP vs molecular weight. B) Scatterplot of Q value vs peptide length. The peptides are colored according to their taste quality (green points for bitter peptides, and blue points for nonbitter peptides). All points represent a canonical peptide of the BPS-1000.

The Molecular Weight (MW) is a commonly used parameter to estimate the size of molecules. The smallest molecular weight is that of the smallest amino acid, G (75 Da), and the highest molecular weight is that of the longest peptide, the nonbitter peptide YPV­QPF­TES­QSL­TLT­DVE­NLH­LPP­LLL­QSW­MHQ­PHQ­PLP­PTV­MFP­PQS­VLSL­SQSK (6357 Da) with its 56 amino acids. Most peptides (754 of 785) describe molecular weights of less than 2000 Da.

In the case of peptides, their size can also be inferred by the number of amino acids (MW and peptide length have a strong positive correlation, Figure S2A). Similarly, the logP (octanol–water partition coefficient) measures molecule’s hydrophobicity, however, Tanford introduced the *Q* value to estimate hydrophobicity specifically for peptides and amino acids.^51^ This value reflects the average free energy of amino acids that describes the transfer of amino acid side chains from ethanol to water (*Q* = ∑Δ*f*/*n*). The correlation between *Q* values and logP values is weaker than that between MW and length (Figure S2B). Therefore, the resulting peptide spaces, calculated with the peptide-specific measures *Q* values vs length and also with calculated logP vs MW, have different shapes (Figure 4).

Both plots show the enrichment of bitter peptides at increasing hydrophobicity values, while there is no clear distinction along the size axis, aligning with the *Q* rule’s principle. The smallest *Q* value represents the amino acid *Q* (−100 cal/mol), and the highest is the dipeptide WW (3000 cal/mol). According to this rule, peptides with *Q* values over 1400 cal/mol are likely to be bitter, while nonbitter peptides have *Q* values below 1300 cal/mol. Applying this rule to BPS-1000 (Figure 4B, dark red separation line at *Q* value 1400 cal/mol), we see that the plot region with *Q* values above 1400 cal/mol is enriched with bitter peptides (green points). We quantified this enrichment with the density distribution of the *Q* values in Figure S3. However, many nonbitter peptides (blue points, 80 peptides) also occupy this high *Q* value region (green points, 349 peptides). The classification becomes less clear in the low *Q* value region, as 102 bitter peptides fall under the threshold of 1300 cal/mol. Interestingly, it seems that the distinction between bitter and nonbitter peptides based on *Q* values appears to blur further as the amino acids’ length increases, suggesting that molecular size might complicate this classification.

The shift of bitter peptides toward increased hydrophobicity is also confirmed by a density plot of logP values (Figure S4). A logP value greater than −0.20 is calculated for 184 peptides, 168 of these 184 peptides are bitter (91.30%). By analyzing the amino acid distribution of these peptides up to a length of 13 amino acids, we found a higher occurrence of the amino acids F, I, L, P, V and Y (Figure S5), confirming the impact of these amino acids within a peptide to develop a bitter taste as already highlighted in our previous work.^23^ However, with decreasing logP values, the distribution of bitter and nonbitter gets more blurred (151 bitter peptides and 210 nonbitter peptides have a logP < −1.90 within the density plot for nonbitter peptides, Figure S5). However, like the *Q* value, the explanatory value of logP is limited due to the missing 3D-structural information of peptides like chirality, intramolecular hydrogen bonds, or long-range interactions that complicate the classification of large peptides.

Then we looked at the bitter peptide space by BTTs (Figure 5). In our database, BTT values range from less than 1 mmol/L to more than 100 mmol/L. It does not seem that there is a correlation between BTTs and *Q* values, and peptides with different bitter intensities are distributed through the peptide space. Most peptides (292) have BTTs lower than 10 mmol/L (86%). The peptide with the smallest BTT is RRPP­PFFF (0.002 mmol/L) with a molecular weight of 1062 Da and a *Q* value of 2151 cal/mol. The peptides with the highest thresholds of 403 and 519 mmol/L are the dipeptides GE and GF, with *Q* values of 275 and 1325 cal/mol, respectively. In general, the BTTs of the bitter peptides shift in the direction of greater *Q* values.

**Figure 5:**
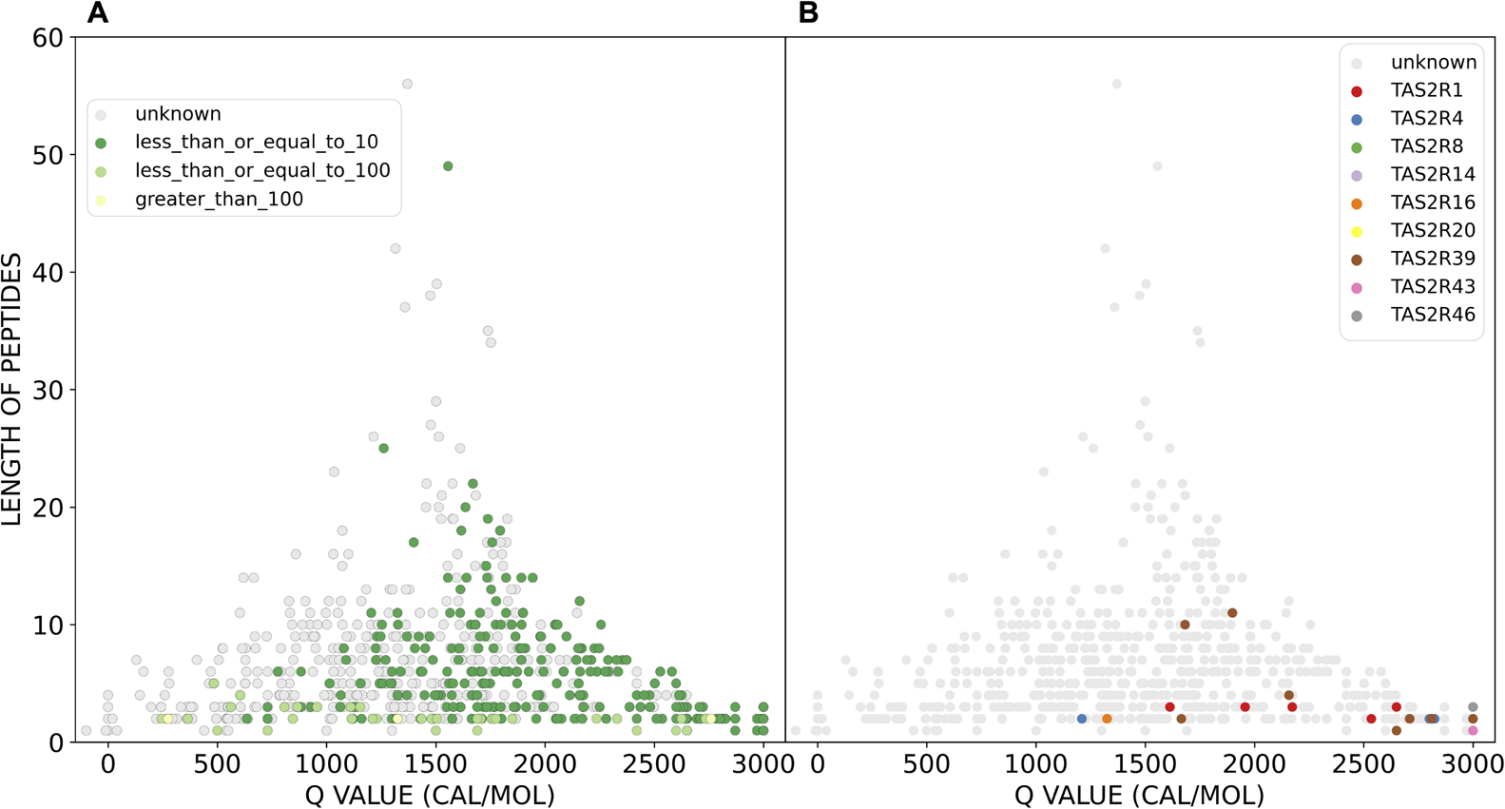
Sensory information and receptor activation of BPS-1000. All points represent a canonical peptide of the BPS-1000. A) Bitter peptides are colored according to their BTTs, from dark green for a lower BTT to light green for a higher BTT. The BBT range of the set spans from 0,002 to 519 mmol/L. B) Bitter peptides are colored by the bitter taste receptors that they activate (data from Table 1). Individual peptides can activate more receptors, the dots in this figure only indicate one activated receptor, while the complete set of receptors can be consulted in Figure S5.

In Figure 5B and Figure S6, we colored the peptides according to the receptor activity, pinpointing the smaller portion of bitter peptides for which we have receptor data. The peptide-sensitive bitter taste receptors found so far are TAS2R1, −4, −7, −8, −14, −16, −20, −38, −39, −41, −43, and −46 (Table 1). The receptor that was found to be activated by the highest number of peptides is the receptor TAS2R1. The lowest activation value was found for the peptide WWW against the receptor TAS2R4. Importantly, Figure 5 underscores the limited number of bitter peptides with corresponding receptor data, highlighting a major gap in our current understanding. Recent breakthroughs in experimental and computational structural biology open new avenues for exploring the bitter taste activity of peptides using structure-based investigations,^52,53^ underscoring the need for expanded receptor-focused studies.

The BPS-1000 also contains 213 modified peptides, comprising 59 cyclic peptides, 14 pyroglutamic acid derivates, 57 γ-glutamyl and γ-aspartyl peptides, 14 norleucine and norvaline derivatives, 26 salts and esters of peptides, plus a group of 43 other peptides, e.g. ornithine-containing peptides or peptides with additional side chains.

Projected in the logP/MW chemical space, modified peptides cluster in the low MW region of the peptide space compared to the wider space occupied by canonical peptides (Figure 6). The peptides of group 7 are a collection of diverse modified peptides, including peptides that contain phosphate, sulfoxide, ornithine, additional side chains or the peptides Bacitracin or YKC­KDX­XLR, and, therefore, occupy a larger region (the green most widespread points in Figure 6). 92 of modified peptides are bitter, while 121 are not bitter. The cyclic peptides and the peptides containing norleucine and/or norvaline or their derivates are mainly composed of bitter peptides (74.6% or 100%, respectively), whereas γ-glutamyl and γ-aspartyl peptides are mostly not bitter (87.7%). Interestingly, the bitter and nonbitter sets of modified peptides are clearly visually distinguished by hydrophobicity (Figure S7). In the context of food applications, there is a strong interest in peptides generated by processing techniques like fermentation (e.g., the usage of citric acid or lactic acid), heating processes or the application of enzymes, e.g. succinyltransferase or pyroglutamylcyclase, to improve the understanding of peptide’s role in human health and its acceptance of consumers in the food industry.^54–56^ The inclusion in our database of modified peptides and the standardized representation for both canonical and modified peptides (*i.e*., HELM and BILN) will serve as a fundamental resource for future investigations.

**Figure 6:**
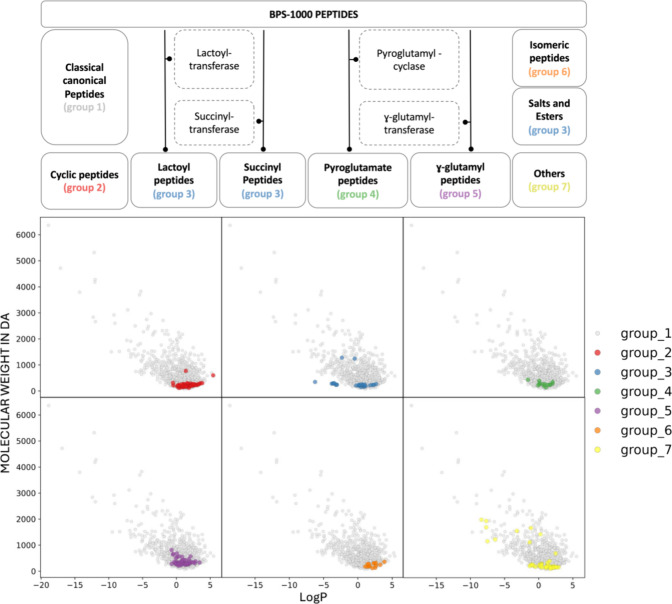
Peptide space of BPS-1000 defined by logP and MW. Coloring of dots follows the classification of BPS-1000 peptides as reported on the right side of the plot: group 1 for canonical peptides, group 2 for cyclic peptides, group 3 for salts or esters including lactoyl- and succinyl-peptides, group 4 for pyroglutamyl peptides, group 5 for γ-aspartyl- and γ-glutamylpeptides, group 6 for any peptide containing norvaline or norleucine and group 7 for other peptides. The grouping of the modified peptides is reported in Table S2.

## Supplementary Material



## Notes

Schematic representation of the methodology (Figure S1), correlations and distributions of physicochemical properties (Figures S2–S4), distribution of more hydrophobic peptides (Figure S5), enhanced visualization of receptor information (Figure S6), peptide space of BPS-1000 with bitterness label (Figure S7), EC_50_ (mM) of peptides activating human bitter taste receptors (Table S1), grouping of modified peptides (Table S2) (PDF)
