# Supplementary material for: Exploring the Molecular Space of Bitter Peptides via Sensory, Receptor, and Sequence Data
Source: J Agric Food Chem. 2025 Jul 23;73(31):19642–51. doi: 10.1021/acs.jafc.5c01195 (PMC12333339; doi:10.1021/acs.jafc.5c01195)
Supplement: Supplementary file 1 [file jf5c01195_si_001.pdf]

Supplementary Information

Exploring the molecular space of bitter peptides via sensory, receptor, and sequence data

Alexandra Steuer[§ab], Laura Sophie Eckrich[§cd], Silvia Schaefer[ab], Verena Karolin Mittermeier-Kleßinger[c], Alexander Otterbach[b], Maik Behrens[b], Corinna Dawid[cd]*, Antonella Di Pizio[be]*

[a] TUM Graduate School, TUM School of Life Sciences, Technical University of Munich, Alte Akademie 8, 85354 Freising, Germany.

[b] Leibniz Institute for Food Systems Biology at the Technical University of Munich, 85354 Freising, Germany.

[c] Professorship for Functional Phytometabolomics, TUM School of Life Sciences, Technical University of Munich, 85354 Freising, Germany.

[d] Chair of Food Chemistry and Molecular Sensory Science, TUM School of Life Sciences, Technical University of Munich, 85354 Freising, Germany.

[e] Professorship for Chemoinformatics and Protein Modelling, TUM School of Life Sciences, Technical University of Munich, 85354 Freising, Germany.

[§] equal contribution

---

*E-MAIL: a.dipizio.leibniz-lsb@tum.de; corinna.dawid@tum.de

Figure S1. Schematic representation of data preparation, cleaning, integration and analyses that led to the BPS-1000 dataset.

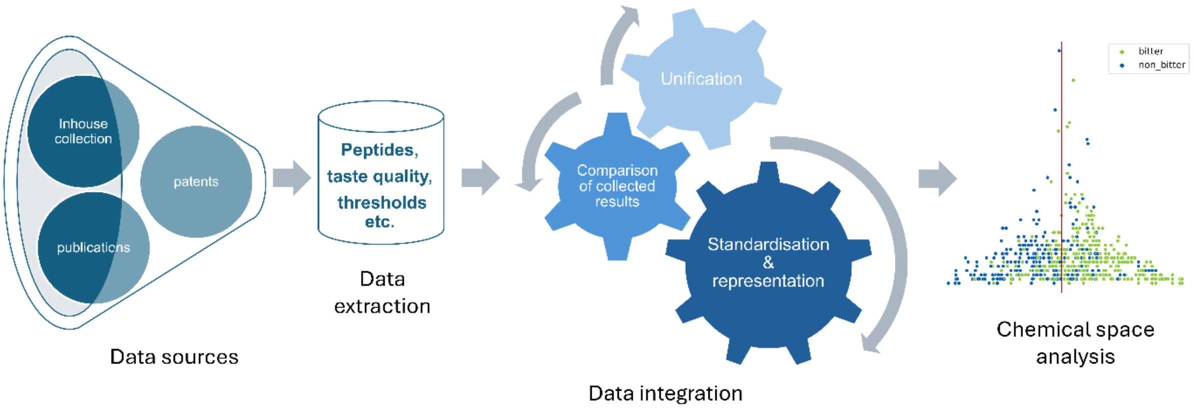

Figure S2. Correlations between size measures (A) and hydrophobicity measures (B) of BPS-1000 peptides. On the left side, green points present bitter peptides, and blue points present non-bitter peptides, Pearson coefficient is reported; on the right side, correlation parameters are reported.

A

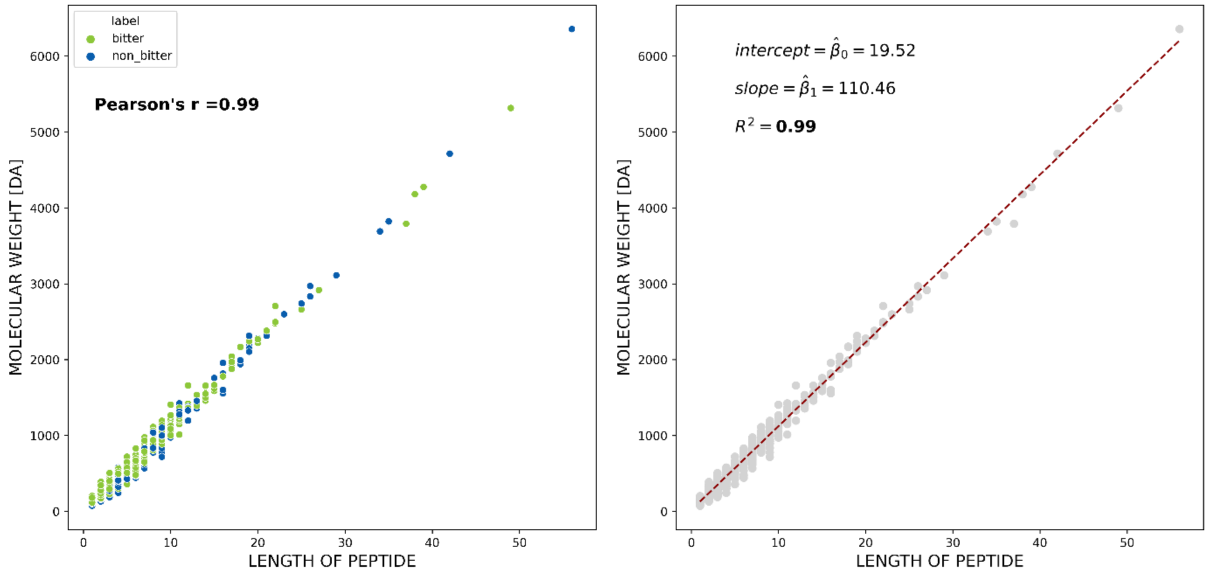

B

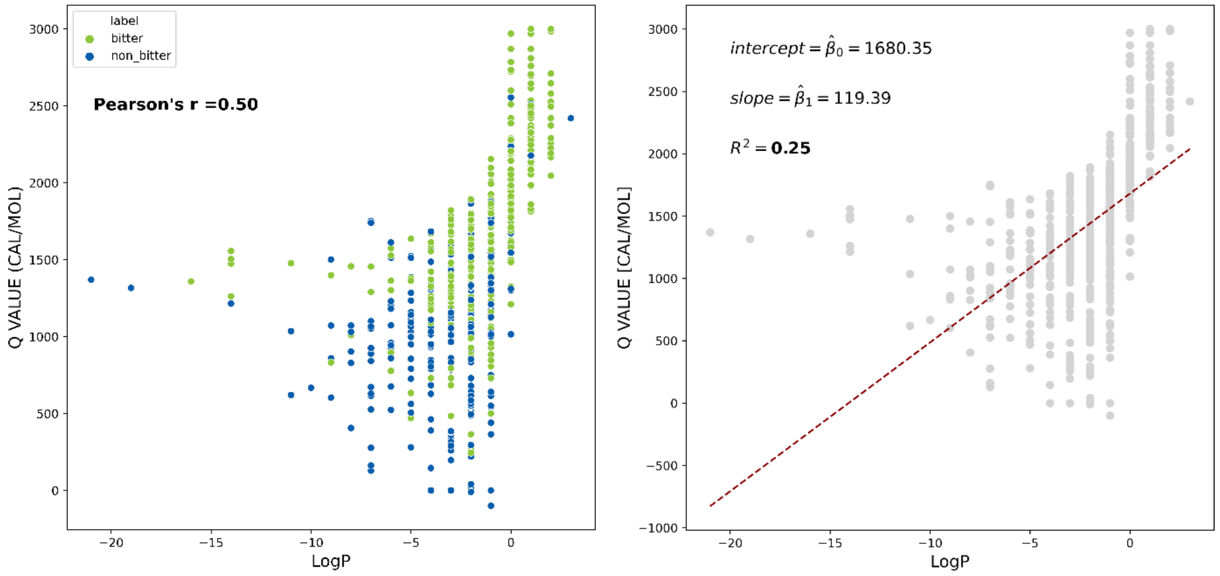

Figure S3. The density plot distribution of Q values. The dark colored dash-dotted line shows the limit of the Q rule (1400 cal/mol). The max. of the density plot for bitter peptides at a Q value of 1707 cal/mol at a maximum density value (KDE) of 0.00081(For non-bitter peptides: Maximum KDE Value: 0.00069 at x = 1078). The lines are colored according to their taste quality (the green line presents bitter peptides, and the blue line presents non-bitter peptides). All points represent a canonical peptide of the BPS-1000.

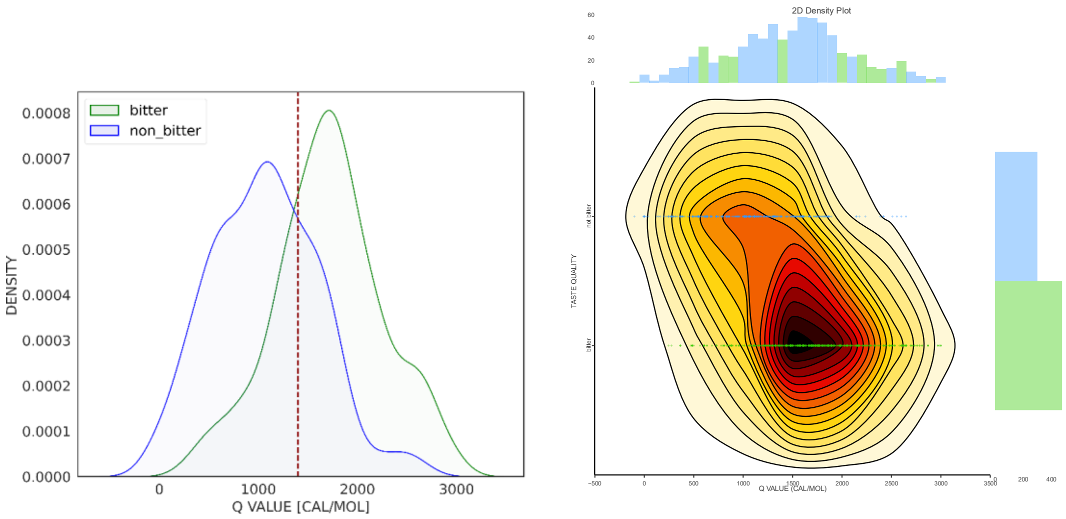

Figure S4. The density plot distribution of LogP values (green line for canonical bitter peptides and blue line for canonical non-bitter peptides). The dash-dotted line (coloured gold-yellow) shows the max. of the density plot for bitter peptides at LogP of -0.20 at maximum density value (KDE) of 0.25 (For non-bitter peptides: Maximum KDE Value: 0.21 at x = -1.90).

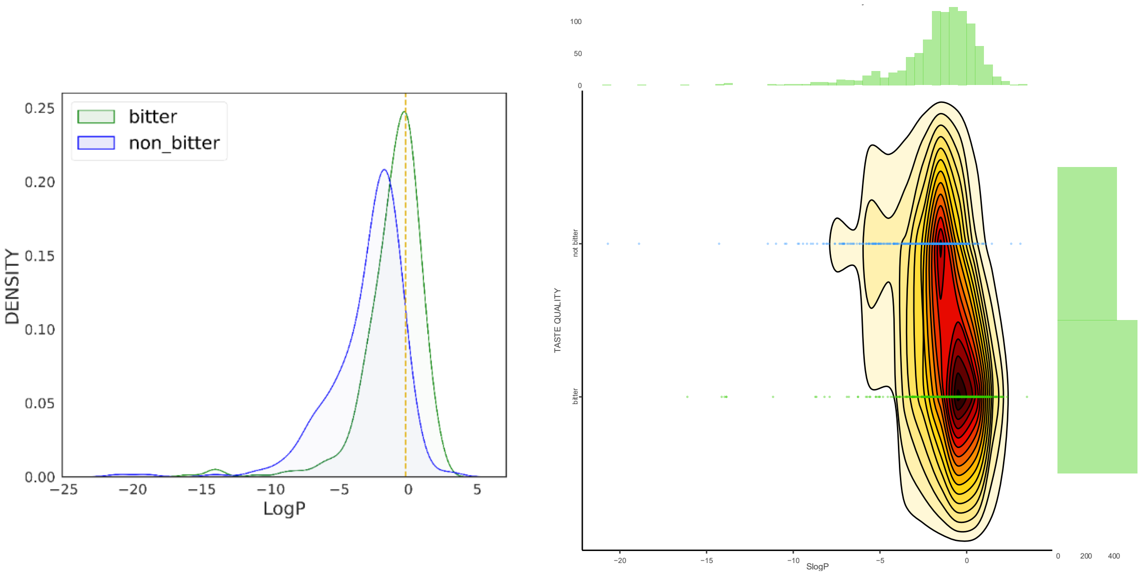

Figure S5. Bar chart distribution of the present number of amino acids of canonical peptides with a LogP ≥ -0,2.

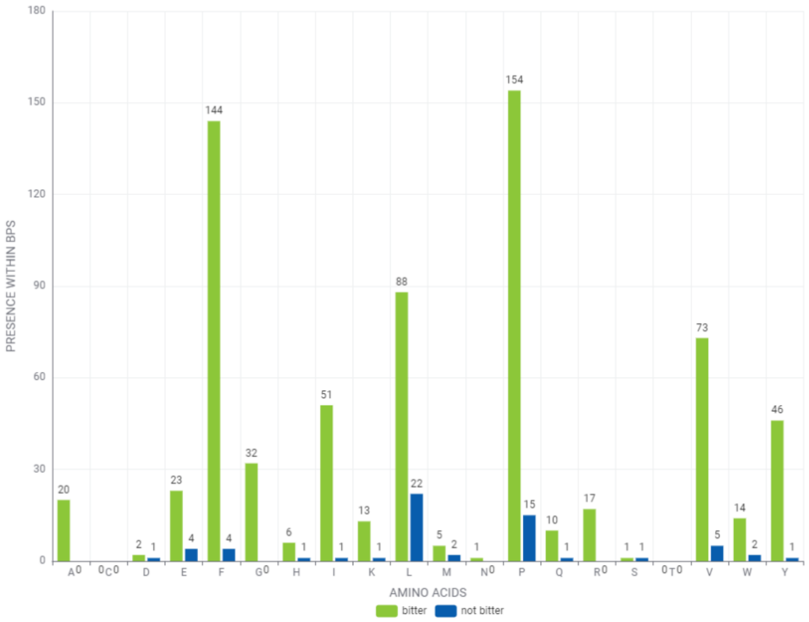

Figure S6. Enhanced swarmplot for visualization of overlaying receptor information. Q values in the x axis, and the length of the peptides in y.

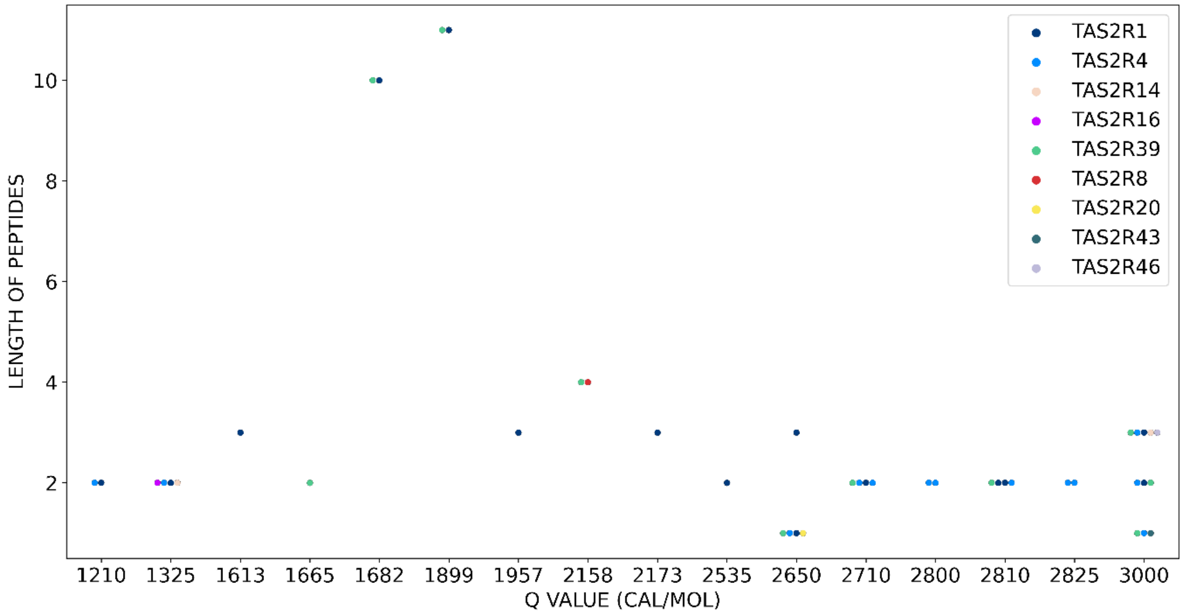

Figure S7. Peptide space of BPS-1000 defined by LogP and MW. The modified peptides are colored according to their taste quality (green points present bitter peptides, and blue points present non-bitter peptides). The classical canonical peptides are represented as greyish points.

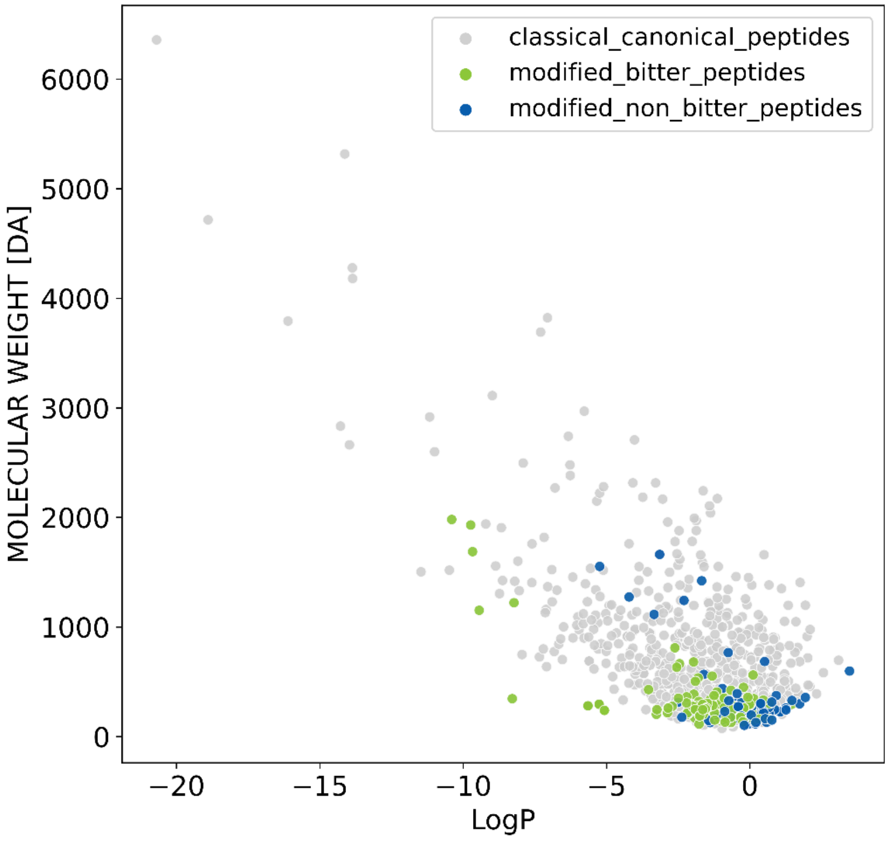

Table S1. EC_{50} (mM) of amino acids/peptides activating human bitter taste receptors.

AMINO ACIDS / PEPTIDES	TAS2R1	TAS2R4	TAS2R8	TAS2R14	TAS2R16	TAS2R20	TAS2R39	TAS2R43	TAS2R46
FFF	0.37								
FFPR			1.1				8.4		
FL	7.2								
GF	7.3								
GL	2.5								
GLL	4.9								
IF	7.4								
IQW	6.1								
LKP	7.6								
WW		0.66							
WWW		0.03							

Table S2. Grouping of modified peptides

Groups	Amino acid/ peptide
**group_2**	[cyclo(AA),cyclo(AF),cyclo(AG),cyclo(AI),cyclo(AL),cyclo(AP),cyclo(AV),cyclo(AY),cyclo(DF),cyclo(DG),cyclo(EG),cyclo(FD),cyclo(FF),cyclo(FG),cyclo(FI),cyclo(FL),cyclo(FN),cyclo(FS),cyclo(FV),cyclo(GF),cyclo(GG),cyclo(GI),cyclo(GL),cyclo(GP),cyclo(HF),cyclo(IG),cyclo(II),cyclo(KG),cyclo(KK),cyclo(L),cyclo(LG),cyclo(LL),cyclo(LP),cyclo(LWLW),cyclo(NF),cyclo(NP),cyclo(Orn-G),cyclo(Orn-Orn),cyclo(PA),cyclo(PF),cyclo(PG),cyclo(PI),cyclo(PL),cyclo(PN),cyclo(PP),cyclo(PT),cyclo(PV),cyclo(PY),cyclo(RF),cyclo(RG),cyclo(RGPPFIV),cyclo(RP),cyclo(RR),cyclo(VF),cyclo(VG),cyclo(VL),cyclo(VV),cyclo(VY),cyclo(WL)]
**group_3**	[(N)-(L)-Lac-(L)-A,(N)-(L)-Lac-(L)-D,(N)-(L)-Lac-(L)-E,(N)-(L)-Lac-(L)-N,(N)-(L)-Lac-(L)-Q,(N)-(L)-Lac-G,(N)-Suc-(L)-A,(N)-Suc-(L)-D,(N)-Suc-(L)-E,(N)-Suc-(L)-F,(N)-Suc-(L)-I,(N)-Suc-(L)-L,(N)-Suc-(L)-N,(N)-Suc-(L)-Q,(N)-Suc-(L)-V,(N)-Suc-G,DL*HCl,EL*HCl,GK*HCl,KG*HCl,KK*2HCl,LD*HCl,LE*HCl,Orn-beta-A*HCl,QLFGPNVNPW-COOC2H5,QLFNPSTNPW-COOC2H5]
**group_4**	[pyroGlu,pyroGlu-A,pyroGlu-E,pyroGlu-EE,pyroGlu-F,pyroGlu-K,pyroGlu-L,pyroGlu-P,pyroGlu-Q,pyroGlu-QAT,pyroGlu-R,pyroGlu-S,pyroGlu-V,pyroGlu-VL]
**group_5**	[A-y-DA,A-y-EA,G-y-DG,G-y-EG,L-y-DL,L-y-EL,V-y-DV,V-y-EV,y-E-((E)-(S)-1-propenyl-C),y-E-((E)-(S)-1-propenyl-C)-((E)-(S)-1-propenyl-C-Sulfoxid),y-E-((E)-(S)-1-propenyl-C)-y-E-((E)-(S)-1-propenyl-C),y-E-((E)-(S)-1-propenyl-C-Sulfoxid),y-E-((S)-allyl-C),y-E-((S)-allyl-C)-G,y-E-((S)-allyl-C-Sulfoxid),y-E-((S)-allyl-C-Sulfoxid)-G,y-E-((S)-ethyl-C),y-E-((S)-ethyl-C)-G,y-E-((S)-ethyl-C-Sulfoxid),y-E-((S)-methyl-C),y-E-((S)-methyl-C)-G,y-E-((S)-methyl-C-Sulfoxid),y-E-((S)-propyl-C),y-E-((S)-propyl-C)-G,y-E-((S)-propyl-C-Sulfoxid),y-E-y-E-y-E-y-E-y-EF,y-E-y-E-y-E-y-EF,y-E-y-E-y-E-y-EM,y-E-y-E-y-E-y-EV,y-E-y-E-y-EF,y-E-y-E-y-EM,y-E-y-E-y-EV,y-E-y-E-y-EY,y-E-y-EF,y-E-y-EM,y-E-y-EV,y-E-y-EY,y-EA,y-EC-beta-A,y-ECG,y-ED,y-EE,y-EF,y-EG,y-EGF,y-EH,y-EI,y-EK,y-EL,y-EM,y-EQ,y-ET,y-EV,y-EVG,y-EVL,y-EW,y-EY]
**group_6**	[(L)-Norleucine or (L)-Nle or Nle,(L)-Norvaline or (L)-Nva or Nva,G-Nle,G-Nle-Nle,G-Nva,G-Nva-Nva,Nle-G,Nle-G-Nle,Nle-Nle,Nle-Nle-Nle,Nva-G,Nva-G-Nva,Nva-Nva,Nva-Nva-Nva]
**group_7**	[(D)-F,(D)-W,(E)-(S)-1-Propenyl-C,(E)-(S)-1-Propenyl-C-Sulfoxid,(L)-2-Aminobutanoic acid,(L)-3,4-Dihydroxy-F,(L)-Ornithine or (L)-Orn or Orn,(L)-Phenylglycine or (L)-Phenyl-G,(L)-Pipecolic acid,(RGPPFIV)2,(RPFF)2,(RPFF)3,(S)-Allyl-C,(S)-Allyl-C-Sulfoxid,(S)-Ethyl-C,(S)-Ethyl-C-Sulfoxid,(S)-Methyl-C,(S)-Methyl-C-Sulfoxid,(S)-Propyl-C,(S)-Propyl-C-

	Sulfoxid,(Z)-(S)-1-Propenyl-C,(Z)-(S)-1-Propenyl-C-Sulfoxid,Bacitracin,D-Orn,DA-OMe,DF-OMe,DG-OMe,E-Orn,EIVPNS[phos]VEQK,G-Orn,K-Tau,KVPQLEIVPNS[phos]AEERL,Orn-D,Orn-E,Orn-G,Orn-Orn,Orn-P,Orn-PPFIV,VP-Orn,VPQLEIVPNS[phos]AEER,YKCKDXXLR,YKVPQLEIVPNS[phos]AEER,YP-Orn]
